# Health services utilization by school going Omani adolescents and youths with DSM IV mental disorders and barriers to service use

**DOI:** 10.1186/1752-4458-3-22

**Published:** 2009-09-25

**Authors:** Asya A Al Riyami, Samir H Al Adawi, Hilal A Al Kharusi, Magdi M Morsi, Sanjay S Jaju

**Affiliations:** 1 Directorate of Research & Studies, Directorate General of Planning, Ministry of Health (HQ), Muscat, Sultanate of Oman; 2 Department of Behavioral Medicine, College of Medicine & Health Sciences, Sultan Qaboos University, Muscat, Sultanate of Oman

## Abstract

**Background:**

Recent corpus of research suggests that psychiatric disorders amongst adolescents and youths are an emerging global challenge, but there is paucity of studies exploring health services utilization by this age group in Arab region.

**Aim:**

This study focus on the health services utilization and the barriers among school going adolescents and youths with DSM IV disorders in the country Oman, whose population is predominantly youthful.

**Methods:**

Representative sample of secondary school Omani adolescents and youths were concurrently interviewed for the (i) presence of DSM IV mental disorders using the face-to-face interview, World Mental Health-Composite International Diagnostic Interview (WMH-CIDI), (ii) tendency for health care utilization and (iii) predictors of utilization with clinical and demographic background.

**Results:**

The proportions of lifetime cases having ever made treatment contact are low, being 5.2% for any anxiety disorder and 13.2% for any mood disorder category. None of these anxiety cases made treatment contact in the year of onset of the disorder, and the median delay when they eventually made treatment contact is about 14 years. In any mood disorders category only 3.6% made contact within the 1st year of onset with the median delay in initial treatment contact is two years for the Bipolar disorder (broad), four years for Any Mood disorder and nine years for the Major Depressive Disorder group. Male gender is significantly associated with less likelihood of making treatment contact when suffering from Social phobia (p = 0.000), Major Depressive Disorder (p = 0.000) and Bipolar Disorder (p = 0.000). The younger cohorts of 14-16 years and 17-18 years of Social phobic made significantly less lifetime any treatment contact (p = 0.000). The 14-16 year olds were significantly less likely to make lifetime any treatment contact for Bipolar Mood disorder (p = 0.000), while the 17-18 group were 1.5 times more likely to do so. Over past 12 months only between 6 to 12% of those having some form of mental disorder avail of any treatment facility with utilization pattern nearly equal between the any healthcare and any non healthcare facilities. In the any healthcare services, more of those with anxiety disorders seek help from general medical doctors while those with Major Depressive Disorder and any Mood disorders are comparatively treated more by non allopathic services. Females were 13.5 times more likely to avail treatment(chi sq 7.1) as also those cases with increased severity of illness were 7 times more likely(chi sq 9.6). In the any treatment category for any 12 month disorder in general, the younger cohort of 14-16 years is 2.2 times more likely to receive any treatment over past 12 months (p = 0.042) while the situation shows marked reversal in the 17-18 age groups. Having any mood disorder is a significant predictor for the same (p = 0.040).

**Discussion:**

Present findings confer with other studies from elsewhere suggesting under utilization of health care services for those with mental illness. Since cultural teaching and traditional coping with mental illness are contributing significantly in furnishing mental health need for many in Oman, the findings are discussed within social-cultural context that forms the basis of the complex health care utilization in Oman. This could foster policies that help bridge the gap between allopathic and non-allopathic care services.

## Introduction

Studies have shown that failure and delays in initial help seeking for mental health problems are a common phenomenon worldwide [1-3] with implication in translating the present distress into more impervious and refractory conditions [4]. In one study comparing the international trend, Wang et al [5] have reported that majority of people with recent episodes of mental illness continue to go untreated. Such situation prevails even in economically advantaged societies or those places where mental health services are readily available [5]. In developing countries where 80% of humanity lives, only a minority who suffers from mental illnesses tend to seek psychiatric treatment under the umbrella of biomedical model [6].

Various barriers to utilization of mental health services in developing countries have been identified including inaccessibility to the services, stigma attached to psychiatric settings [7,8] and the largely ignored factor that many nonmedical treatments including traditional healings [9] are perceived in many societies to integrate distressed individuals back to the community [10]. It has been estimated that 40-90% of the population in developing countries use traditional medicine for their health needs [11]. This figure is likely to be much higher in rural areas where the overall health resources furnished by modern medicine still remain rudimental. There is anecdotal evidence that doctor shopping is very common: sometimes both modern and traditional health systems are concurrently utilized [12]. The situation is compounded by reality on the ground that majority of the population dwells in the urban region where psychiatric settings are poorly served. Approximately 680 million people from developing countries have no access to biomedical mode of psychiatric care [13].

While the psychiatric services in developing countries have much to be desired [14], there is hope for optimism in many quarters as there is some evidence suggesting emerging acceptance of biomedical psychiatric services [8,15]. To our knowledge, there is paucity of studies exploring the utilization of psychiatric services in certain age groups in developing countries. Such dataset could be used to lay groundwork for enlightened policies, allocation of resources and designing intervention and prevention programs for the emerging global increase in mental illness amongst the young [16].

Oman presents an interesting ground for exploring utilization of psychiatric services. Its population structure is pyramidal with majority in adolescence, an age group known to be vulnerable for emotional turmoil and, if their psychological distress is not mitigated then it is likely to be associated with more functional impairment, chronicity and poor quality of life [17,18]. Oman's population of approximately 3.5 million has a preponderance of children and adolescents, with 42.7% of its inhabitants being under the age of 15. The median age of the total population is 18.8 years, it being 21.1 years for males and 16.7 years for females [19]. An unprecedented rise in the standard of living has shielded young Omanis from much of the traditional stresses of growing up, typical for schoolchildren in developing countries [20] though preliminary studies are showing that the adolescent psychiatric disorders are emerging to be common occurrence [21]. One of the major benefits of the recent modernization in Oman is the improvement in living standards and the establishment of modern health care delivery systems with the government providing all its Omani citizens a universally free access to the allopathic health care services [22]. As in many traditional communities, modern psychiatric care has yet to play dominant role in delivery of service for people with psychiatric illnesses. What was deemed as 'mental illnesses' was largely in the appanage of individual dispensing non-biomedical interventions [23] with implication for accessibility, diagnosis and prognostic indicators [24]. As the 'psychiatric disorders' are perceived as nonmedical idioms of distress including supernatural forces such a *Jinn*, contemptuous envy (*Hassad*), the envy-related 'eye' (*Ain*) and sorcery (*Sihr) *[23], there is little evidence to suggest pervasive and persistent stigma toward people with mental illness in traditional Omani society[25]. In recognition with emerging rising tide of mental illness, the Ministry of Health, the main provider of health care in Oman, had initiated program for primary health workers to recognize psychiatric disorders [26]. However, anecdotal report suggests that patients under utilize such facilities [27]. Therefore exploring this issue among adolescents and youths would be essential. With the recent spread of education, the increased affluence and narrow gender gap among younger generation in Oman, studies are needed to shed light on the utilization of emerging psychiatric services in the country.

Previous reports have focused on perceived need for mental health care among Arab population, mostly on adult population [15,28] and using self-related measure[6,28] with all the limitation this may entail. In order to take this issue further, this study aims to explore mental health services utilization by a representative sample of adolescents and youths suffering from DSM IV disorders detected using the face-to-face WMH-CIDI. The specific aim of this study is to explore the treatment contact ever made for mental problems by sufferers and the sociodemographic predictors (sex and age) for lifetime treatment contact, the access and utilization of health services in the past 12 months, the delays that currently exist and the predictors of those delays. Ages of onset for individual disorders and the ages of first treatment contact for each disorder were used to determine the failure to and the delay in access.

## Methodology

### Samples and diagnostic assessment

This study was the second phase of the two-stage epidemiological survey conducted in 2005. The respondents in both study phases were between 14 and 23 years of age [29]. The survey adopted a nationally representative, multi-stage, stratified random sampling design to select its subjects. The latest data obtained from the Ministry of Education, Oman was used as the sampling frame [30]. The sample was weighted (Table 1) based on the number of Omani secondary school students from all the different regions of Oman, their gender distribution, their classes ranging from grade 1 to grade 3 and their stream of study [31].

**Table 1:** Sociodemographic distribution of the study sample compared to population of secondary school Omani students.

	**P1 Unweighted**		**P2 Unweighted**		**P1 Weighted**		**P2 Weighted**		No. of students*	
**Sex**	**N**	**%**	**N**	**%**	**N**	**%**	**N**	**%**	**N**	**%**
	
Male	832	49.5	238	47.0	794.6	47.2	239.5	47.2	52167	47.2
Female	850	50.5	269	53.1	887.4	52.8	267.5	52.8	58253	52.8
										
Class No										
Grade 1	675	40.2	174	34.34	679.5	40.4	204.8	40.4	44610	40.4
Grade 2 -- Art	270	16.1	79	15.58	234.3	13.9	70.6	13.9	15382	13.9
Grade 2 -- Science	293	17.4	97	19.14	297.7	17.7	96.8	19.1	19544	17.7
Grade 3 -- Art	216	12.8	68	13.42	214.7	12.8	57.7	11.4	13061	11.8
Grade 3 -- Science	228	13.6	89	17.55	255.7	15.2	77.1	15.2	17823	16.1
										
Region										
Muscat	277	16.5	75	14.8	324.6	19.3	97.8	19.3	21306	19.3
Dhofar	197	11.7	71	14.0	153.1	9.1	46.1	9.1	10049	9.1
Al-Dakhliyah	258	15.3	82	16.2	238.6	14.2	71.9	14.2	15666	14.2
N Sharqiyah	134	8.0	52	10.3	117.1	7.0	35.3	7.0	7688	7.0
S Sharqiyah	140	8.3	41	8.1	123.9	7.4	37.3	7.4	8132	7.4
N Batinah	366	21.8	110	21.7	367.4	21.8	110.8	21.8	24121	21.8
S Batinah	193	11.5	42	8.3	223.5	13.3	67.4	13.3	14676	13.3
Al-Dhahirah	117	7.0	34	6.7	133.8	8.0	40.3	8.0	8782	8.0
										
Totals	1682	100.0	507	100.0	1682	100.0	507	100.0	110420	100.0

* source Ministry of Education data 2002

In the first phase (phase 1), a screening of 2,739 male and 2,670 female adolescents and youths (total n = 5,409) with the General Health Questionnaire (GHQ-12) and the 27 item Child Depression Inventory (CDI) was conducted.

The second phase (phase 2), which is reported here, studied a random sub-sample of 1,836 respondents from the above sample of which 1,682 (91.61%) agreed to participate in the face-to-face structured interview using the Arabic version of WMH-CIDI, PAPI (Paper and Pencil Instrument) version 3.0 [32]. This version was not validated but was professionally translated into Arabic based on a five-step process of forward translation, back translation, resolution of discrepancies between translation and back translation, pilot testing, and final revision. Training for the school health doctors (interviewers) was conducted by certified CIDI qualified trainers as described elsewhere [33]. The Ministry of Education sent circulars to the sampled secondary schools to ensure their participation. The purpose of the study, its methodology and the consent forms supplied by the researchers were appended with the circular. The respective schools obtained written consent from the parents of the sampled respondents. It was clarified that the participation of their child, even after selection, was not compulsory and the parents had the choice to refuse enrollment. They were assured of confidentiality of the findings if their child was selected to be interviewed. Prior to the actual interview, a verbal consent was obtained from the respondent. The Ethics Committee of the Omani Ministry of Health approved the study.

The CIDI was administered in two parts. Part 1 included a DSM IV core diagnostic assessment screen (Part 1 sample (P1): n = 1,682; males = 832, females = 850). Part 2 included questions about risk factors, consequences, and other correlates (health service utilization, adequacy of treatment) along with assessments of additional disorders that were administered to all Part 1 respondents who met lifetime criteria for any disorder. This formed the Part 2 (P2) sample consisting of 507 respondents; males = 238, females = 269.

## Terminology

### Treatment contact

The respondents who answered the following two questions [34] were included in the lifetime treatment contact analyses: Did you ever in your life talk to a medical doctor or other professional about your existing problem? And how old were you the first time you talked to a professional about your problem? The interviewer clarified that the term 'other professional' was meant to apply broadly to include Counselors, Social workers in mental health setting, Psychologists, Spiritual advisors, Healers, Chiropractors and any other mental health (healing) professionals (Table 2) [31]. The response to the second question was used to define age of first treatment contact. Service use was defined as a) the proportions of lifetime cases having ever made treatment contact with any of above b) those who made treatment contact in the year of onset of the disorder. The age of onset of mental disorders used to calculate the treatment contact in the year of onset of the disorder is detailed elsewhere [35]. It is based on retrospective reporting. It may be recalled incorrectly. The age of onset focuses on the onset of the syndrome, ignoring any prodrome at an earlier age.

**Table 2:** Classification of the services used for analysis for 12 month treatment.

**Service**	**Includes**
Other mental healthcare	Counselors/Social worker in mental health setting/Psychologists/other mental health professionals
Any mental healthcare	Other mental healthcare + Psychiatrist
Any Healthcare	Any mental healthcare + General Medical(family doctor or other medical doctor)
Human services	Spiritual advisor
Complementary Alternative Medicine (CAM)	Healer/Chiropractor etc
Any Non Healthcare	Human services + CAM
Any Treatment*	Combines Any Healthcare and Any Non-Healthcare*

*Any Treatment is NOT addition of any healthcare and any non-healthcare. It means that did the respondent see any professional from these categories in his/her lifetime and/or past 12 months and/or how many visits were made over past 12 months.

### Initial treatment contact

The durations of delay in initial treatment contact is defined in median years from disorder onset to first treatment contact among cases that eventually made treatment contact [34].

### Predictor variables of treatment contact

Due to sparse data [34], only age cohort (age at interview) and sex are used as predictors of lifetime any treatment contact among respondents with a given lifetime disorder, while the disorder type has been used in addition to the above two predictors for 12 month service usage of any treatment contact. For twelve month treatment contact, age cohort (age at interview), sex and severity of illness were the predictors.

### Severity of illness for 12 month treatment contact [36]

Respondents who met WMH-CIDI criteria for a lifetime diagnosis and who reported recency within the past 12 months were assessed for the 12-month severity of their disorder based on the following criteria.

Severe: if respondent is diagnosed with 12-month Bipolar I disorder, if respondent has attempted suicide in last 12-months and had any 12-month diagnosis or if respondent has more than one 12-month diagnosis and high level of impairment on the Sheehan disability scales.

Moderate: At least one 12-month disorder and moderate level of impairment.

Mild: any 12-month disorder.

### Collapsing rows and columns [37]

Any Mood disorder, Any Anxiety disorder and Any Impulse Control disorder are aggregated categories of the disorder. They are created by collapsing the rows and/or columns in the tables for that particular category when there are too few respondents with individual disorders in that particular category. Similarly if too few respondents have used particular service sectors, the columns for individual sectors have been collapsed and reported as 'Any Mental Health Specialty', 'Any General Medical', 'Any Non-Healthcare' and 'Any Treatment'.

### Statistical analysis

The statistical analysis has been done by the Department of Health Care Policy, Harvard Medical School, Boston, USA using the Statistical Analysis Software program and the same statistical methodology used in the previously reported CIDI studies [[34,36], and [37]]. Hierarchy is not used in any disorder. Survival curves are used to make projections of the proportion of cases that will eventually make lifetime any treatment contact for each disorder assessed. All the eligible respondents have been observed for a period of 20 years from the year of onset of the disorder. Predictors of treatment contact were examined separately for each disorder using discrete-time survival analysis with person year as the unit of analysis. Wald chi-square test using Taylor series design-based coefficient variance-covariance matrices are used to make multivariate significance tests in the discrete-time survival analysis. The analyses follow the guidelines which are based on previous studies [[34,36], and [37]].

## Results

Table 1 looks at the distribution of post stratification variables both unweighted, weighted and in the census population of secondary school Omani students. The sex distribution of the part 1 (P1) sample is within 2% of the census distribution. The same is noted for the Grade 2 Art and Grade 3 Science. Region wise this discrepancy is a maximum of up to 3%. The sex distribution for the part 2 (P2) sample is nearly similar to that of the census. The distribution is the same as that of census for the Grade 2 Science and North Batinah region while discrepancy ranging from 1 to 6% is noted in other categories. Overall the random sub-sample fairly matches the census distribution and can be considered as a representative sample.

The access to health services is reflected in the proportions of lifetime cases having ever made treatment (Table 3) contact is 5.2% if the respondent is classified as having any anxiety disorder. The estimates for the sub groups are 11.1%for Social phobia and 3.9% for Specific phobia. None of these anxiety cases made treatment contact in the year of onset of the disorder, and the median delay when they eventually made treatment contact is 13 years for Specific phobia and 14 years in the Social phobia and Any Anxiety group.

**Table 3:** Treatment contact ever made, made in the year of the onset of disorder and median duration of delay among cases that subsequently made treatment contact.

	**% Ever having** **treatment contact**	**% Making treatment contact** **in the year of onset of disorder**	**Delay in making treatment** **contact (median years)**	**N**
**Anxiety disorders**				
Specific phobia	3.9	0.0	13.0	101
Social phobia	11.1	0.0	14.0	27
Any Anxiety disorders	5.2	0.0	14.0	61

**Mood disorders**				
MDD	11.4	4.8	9.0	53
BMD (broad)	17.8	0.0	2.0	17
Any Mood disorders	13.2	3.6	4.0	69

The results are comparatively better when one had any mood disorders where 13.2% made treatment contact though only 3.6% of these made so within the 1st year of onset. Of the 11.4% MDD who ever made treatment contact, only 4.8% made contact for treatment within the first year of onset of the disorder while of the 17.8% with BMD none did so in the first year of onset. The median delay in initial treatment contact is two years for the BMD (broad), four years for Any Mood disorder and nine years for the MDD group.

Male gender (Table 4) is significantly associated with less likelihood of making treatment contact when suffering from Social phobia (p = 0.000), MDD (p = 0.000) and BMD (p = 0.000).

**Table 4:** Sociodemographic predictors for lifetime treatment contact.

	**Sex**				**Cohort (age of interview)**						
	**Male**				**Age 14-16**		**Age 17-18**		**Age 19+**		
	**OR**	**95% CI**	**Wald Chi sq (df = 1)**	**P**	**OR**	**95% CI**	**OR**	**95% CI**	**OR**	**95% CI**	**Chi sq (df),** **P value**
**Anxiety disorders**											

Specific phobia	0.4	0.0-3.9	0.7(1)	0.400	0.4	0.0-6.1	0.2	0.0-2.6	1.0	1.0-1.0	1.6(2),0.444

Social phobia	**0.0**	**0.0-0.0**	**51.3(1)**	**0.000**	**0.0**	**0.0-0.0**	**0.0**	**0.0-0.0**	**1.0**	**1.0-1.0**	**157.3(2),0.000**

Any Anxiety disorders	0.1	0.0-1.1	3.5(1)	0.063	2.8	0.3-30.5	0.6	0.1-5.0	1.0	1.0-1.0	3.3(2),0.196

**Mood disorders**											

MDD	**0.0**	**0.0-0.0**	**117.6(1)**	**0.000**	2.3	0.3-19.3	1.6	0.2-13.6	1.0	1.0-1.0	0.6(2),0.736

BMD(broad)	**0.0**	**0.0-0.0**	**194.9(1)**	**0.000**	**0.0**	**0.0-0.0**	**1.5**	**0.1-25.8**	**1.0**	**1.0-1.0**	**76.2(2),0.000**

Any Mood disorders	0.6	0.1-5.5	0.2(1)	0.684	1.7	0.2-12.9	1.4	0.2-7.8	1.0	1.0-1.0	0.3(2),0.864

The younger cohorts of 14-16 years and 17-18 years of Social phobic made significantly less lifetime any treatment contact (p = 0.000)while no differences existed across the age groups for the other anxiety disorders. The 14-16 year olds were significantly less likely to make lifetime any treatment contact for BMD (p = 0.000), while the 17-18 group were 1.5 times more likely to do so.

The Table 5 shows the poor service usage over past 12 months by those who require it. Only between 6 to 12% of those having some form of mental disorder (standard errors not considered) avail of any treatment facility as seen from the last column of Table 5.

**Table 5:** 12-month service usage in Oman [approximate percent from among those having mental disorder being treated by different sectors].

	**Any healthcare(Std.error)**	**Any Non Healthcare(Std.error)**	**Any treatment (Std.error)**
Social phobia	12%(7%)	5%(5%)	12%(7%)
Specific phobia	6%(3%)	4%(2%)	6%(3%)
Any Anxiety disorder	7%(3%)	4%(2%)	7%(3%)
MDD	12%(6%)	5%(3%)	12%(6%)
BMD	7%(7%)	7%(7%)	7%(7%)
Any mood disorder	10%(4%)	5%(3%)	10%(4%)

The data show that the pattern of service utilization is approximately equal (considering the overlap due to the standard errors) between the any healthcare and any non healthcare facilities by among those who require it. This percentage of population is however very low, thus suggesting that approximately 88% of those in need of services are not availing it. Even amongst the any healthcare services, more of those with anxiety disorders seek help from general medical doctors while those with MDD and any Mood disorders are comparatively treated more by other mental healthcare. The mean number of visits in any treatment facility amongst those in part 2 sample is 2.4(s.e = 0.6, n = 12) while the same for any healthcare is 2.4(s.e = 0.5, n = 11).

As depicted from Table 6 (standard errors not considered), over the past 12 months, more respondents with Anxiety disorders (Agoraphobia w/o panic + Social phobia) are catered to mainly by the General Medical professionals. The MDD and Any Mood disorders are comparatively treated more by the other mental healthcare (non-medical personnel) than the psychiatrists.

**Table 6:** 12-month service usage in Oman [approximate percent from among those having mental disorder being treated under Any Healthcare facilities]

**Any Healthcare (Std.Error)**			
**Mental disorder**	**Psychiatrist**	**Other mental healthcare**	**General Medical**

Agoraphobia w/o panic	-	-	7%(7%)
Social phobia	7%(5%)	5%(5%)	10%(7%)
Specific phobia	3%(2%)	3%(2%)	3%(2%)
Any anxiety Disorder	3%(2%)	3%(2%)	3%(2%)
Major Depressive Disorder	2%(2%)	9%(5%)	0%(0%)
Bipolar Disorder	7%(7%)	7%(7%)	7%(7%)
Any Mood disorder	5%(3%)	9%(4%)	2%(2%)

The same cohort was analyzed for 12 month healthcare treatment (Table 7). Females were 13.5 times more likely to avail treatment and this is statistically significant. The odds were also significant and showed linear increasing trend as the severity of illness increased. There is no association between age group and utilizing the healthcare treatment. But in the any treatment category (Table 8) for any 12 month disorder in general, the younger cohort of 14-16 years is 2.2 times more likely to receive any treatment over past 12 months (p = 0.042) while the situation shows marked reversal in the 17-18 age groups. Having any mood disorder is a significant predictor for the same (p = 0.040).

**Table 7:** Correlates of 12- month Any Healthcare treatment.

	**OR**	**95% CI**
**Sex**		
Male	1.0	...
Female	13.5	2.0-91.8
Chi square	**7.1**	
**Severity**		
Severe	25.0	2.9-215.9
Moderate	15.9	2.1-118.5
Mild	17.6	1.2-259.2
None	1.0	...
Chi square	**9.6**	

**Table 8:** 12 month service usage in Oman.

**Model effect**	**Effect level**	**Any treatment given** **(Any 12 month disorder)**
Age	14-16	2.2(0.3-15.3)
	17-18	0.2(0.0-3.3)
	19-23	1.0(1.0-1.0)
	Overall test of effect	Wald Chi sq = 6.3, df2, **p = 0.042**
Any Anxiety	Yes	3.3(0.3-39.0)
	No	1.0(1.0-1.0)
	Overall test of effect	Wald Chi sq = 0.9, df1, p = 0.338
Any Mood	Yes	7.7(1.1-54.0)
	No	1.0(1.0-1.0)
	Overall test of effect	Wald Chi sq = 4.2, df1, **p = 0.040**
Sex	Male	0.1(0.0-1.2)
	Female	1.0(1.0-1.0)
	Overall test of effect	Wald Chi sq = 3.3, df1, p = 0.06

Sociodemographic and disorder type predictors of any treatment given.

## Discussion

It is well known that quality of life for people with mental disorders could be dramatically improved, but provision for such services have yet to be made available globally[38]. This has sparked recent calls to improve provision for mental health as there is intricate relationship between mental health despite their amorphous nature and quality of life and the tendency for mental illness to heighten other physical diseases or vice-versa [39].

The bulk of the population Oman is youthful and has borne out all the privileges of modernity including free for all in education and health services. The present discourse has aimed to tease out whether recent affluence has transformed young Omani perceive their distress using odium of modern psychiatry and, if that is the case, it would be interesting to explore whether these youngsters seek help from psychiatric setting. On the whole, this study suggests that the mental health services access is poor for those with marked psychiatric disorders and the pattern of service utilization is approximately equal between the any healthcare and any non healthcare facilities by among those who require it. Such trend is not unique to Oman [40]. Psychiatric fraternity has so far made little intimate relationship with people from developing countries because, on one hand, its presence is hardly visible and, on the other, its conceptual framework is perceived to be incompatible with discursive processes that are inescapable social [41]. In traditional Omani society something akin to mental illness is often viewed as manifestation of spirit possession or fate. The present undertaking is unique since it elicits access and utilization of psychiatric care among school children who are already suffering from mental illness diagnosed with *sine quo *of modern psychiatric, DSM IV. Albeit indirectly, this study highlights viability of diagnosing cross-cultural population using diagnosis tools derived mostly from Western population which is a much criticized feat alleged to reificate nosological categories that are incompatible with the situation on the ground.

The present study suggests that female gender was significantly associated with higher likelihood of making initial treatment contact for lifetime treatment and also over past 12 month period. This collaborates with findings in other countries [5]. However, the situation is complex from the data emerging from Arab/Islamic countries. In clinical setting, the males tend to outnumber females in psychiatric care seeking which could be attributed to the increased severity in males [42] However, in community surveys, there is evidence that women tend to have high propensity to some mental illness [43] though their presentation may be 'masked' due to certain socio-cultural factors [44]. In paternalistic society, male in their public front are sanctioned not to appear weak. Psychological disorders such as mood disorders possession are often perceived to be due to 'weakness of character' or manifestation of possessing spirit. For psychiatric services to take firm root in this part of the world, public education on mental health would be essential. With the background that they tend to be underutilizing the services, the males ought to be aggressively targeted.

It would be theoretically interesting to explore whether there is difference in access and utilization of any treatment (table 2) among different strata of adolescents and youths. This study has explored this issue. The younger cohort of 14-16 years in this study significantly made less lifetime treatment contact for the specific disorders of social phobia and bipolar disorder. However this same group when labeled as having any 12 month disorder was more likely to utilize some form of service when assessed for a 12 month period. This discrepancy is due to considering any diagnostic label over past 12 months rather than the specific category wise analysis in the lifetime treatment. The lack of awareness among parents and respondents or their unwillingness in spite of being aware or poor health education on this issue by concerned authorities are the reasons we can attribute. Our results need to be interpreted with caution due to the narrow age range of our sample. In other studies the younger cohort of 18-34 years was more likely to avail of treatment as compared to the older cohorts [5]. Our 17-18 group having mood disorder have indeed better accessed the treatment when suffering from Bipolar disorder.

The present study implies that psychiatrist services are very poorly utilized but there are several reasons that may be worthwhile to speculate. In the Ministry of Health, Oman, as of the end of the year 2007, there are 34 registered mental health professionals in a population of nearly three and a half million people scattered around 300,000 square kilometers[45]. Most of the services are dispensed within general tertiary hospital, plus two specialized centers located in the capital. According to official algorithm of allopathic care for people with mental illness, the first point of contact will be primary health setting. Most common psychotropic medications are readily available in the primary health care [46]. However, due to the lack of awareness and suitable health education and a lack of a close and relatively informed and consistent relationship with health care givers, people with mental illness may under utilize such option [46]. Instead, there is tendency to seek primary care at tertiary centers. Apart from the teaching hospital, the multidisciplinary infrastructures often essential for psychiatric service have remained rudimental due to lack of suitable manpower. Psychiatric services 'fashion' itself to follow Anglo-American model with strong neurogenetic determinism conceptual outlook and clinical framework. Another factor that may contribute to under utilization of the allopathic services may be related to perceived status of traditional healers. By virtue of their intricate knowledge of social dynamism of their respective society, traditional healers are likely to be perceived in the eyes of community as offering intervention that is compatible with their view of socio-cultural teaching [11]. Another factor that is likely to have direct bearing on the patterns of utilization is with the cultural value system in Oman. Traditional value systems in Oman are those that have affinity to collectivistic orientations [20]. In cultural setting, children are brought up in an environment that ushers them into collective mindset, as development of selfhood is generally discouraged, so is expression of emotions. Depending on the level of education, difficulties are attributed to sensate agents and distresses are communicated in "somatopsychic" rather than "psychic" ways. As traditional Omanis attribute ill-health to external agent, there would be interrelated implications for psychiatry. When a social distress occurs, an individual is likely to frame his or her difficulty to external forces *Jinn*, *Hassad*, *Ain *or *Sihr*. It is not surprising that many psychiatric problems are first attended by traditional healers who are sought to exorcise to malevolent spirit. In term of health policy, there is need to contemplate how to integrate allopathic and non-allopathic approaches for the wellbeing of people with mental disorders as seen from the findings of this study. It has reported that 60% of outpatients at the university have sought advice from traditional healers before coming to the psychiatrist [47]. In support of this view, it has been common observation that the first point of contact among people with any psychological distress is via traditional means, including reading Koran and exorcism [48]. However, following a prostrated 'healer shopping', if their condition is intransigent and impervious to traditional healing systems, the next set of recourse, which is freely available in Oman, is via the extensive network of allopathic health care system. There is also a strong possibility that that sufferer of mental illness may concurrently transverse between these two health care systems. However, since this aspect could not be disentangled in our analysis, it complicates the issue of access of service and pattern of utilization.

Other contributing factors may be socio-cultural patterning. In it well known that in many traditional societies, distress that are akin to psychiatric disorders are not perceived in psychiatric parlance, as intra-psychic conflict. Therefore distress is not viewed to be precipitated by trajectory of one's behavior and one's self and intervening environmental conditions. As psychiatric attempt to heal the 'self', in a society where development of the self is not recognized, it is difficult to see how 'self', as conceived in western psychology, would allow itself to be healed if it does not exist in the first place. This view, within the present context, constitutes one of the reasons that may mitigate poor mental health service utilization which in Oman is based on the exclusive western allopathic model. More studies to collaborate the factors leading to under utilization of mental health services are therefore warranted.

### Limitations

It is essential for us to highlight some of the inherent limitations of these types of studies. School based rather than community surveys have inherent limitation. Firstly, it is possible that there are some students who were either absent or not in mainstream education because of their mental illness and therefore not present in the schools surveyed. Secondly, this study suggests that the mental health services utilization is low. It is possible that during the initial stages of mental illness the signs and symptoms are often quite non specific making it difficult for the sufferers to realize that they are in need of help [34]. It is also possible that many sufferers of mental illness may not seek medical attention because the disorder may be construed as an act of fate or may be simply hidden to avoid social stigma. Thirdly, it is essential to consider the potentially biased recall of mental health service use as a limitation in this study. Mood disorders, by their very nature, come to be noticed earlier and this accounts for the relatively increased proportion making contact in the year of onset of MDD and any mood disorder. This also hold true for 12 month service usage of availing any treatment amongst those having any mood disorder [34]. Another obvious limitation of this study is that it relies heavily on diagnostic tool. Although all items in the assessment measures were translated to achieve conceptual equivalence in the Omani dialect, their usefulness could still be hampered by certain subtle linguistic and conceptual misunderstandings which might not have been apparent during translation and piloting [49]. This is pertinent to the view that there is no universal phenotypical presentation of emotion. Future studies should carefully address the issue of culturally sensitive measures. Ecological validity of the diagnosed psychiatric disorders needs to be considered with caution. Proper diagnosis of minors often requires observations across range of social situations [50]. For example, quantification of many childhood disorders would require opinion from both parent, teachers and as well as information garnered during the interview.

### Suggestions

Despite above-mentioned caveats, it is essential to highlight some important implication of the present findings. They have a direct bearing on the formulation of policies for mental illness in Oman. First of all, despite all the criticisms that have been trumpeted on using western diagnostic tools, these are only available tools that render international comparison. Studies are needed in the Oman on the heuristic value of some of the diagnostic categories on Omani population. Secondly, by virtue of being in society in transition while attempting to transverse modernity and tradition, effort should be underway to formulate culturally sensitive intervention for its youthful populations. It is possible that the sheer increase of this segment of the population is likely to result in proportional increase the number of people who are likely to succumb to mental illness. Thirdly, existing teaching establishments can be utilized to create a cadre of psychologists, psychiatric social workers and other mental health workers, at a fraction of time and money spent to train psychiatrists, to dispense culturally sensitive needs of the emerging population. Fourthly, bearing the cultural teaching and the fact that traditional coping with mental illness are contributing significantly in furnishing mental health need for many in Oman, policies are needed bridging the gap between allopathic and non-allopathic care services. This has always been a contentious issue. Religious and medical mental health care systems have existed for a long time in the West as well as in the East. Although in the past their relationship was characterized by suspicion and conflict, recently many experts have suggested that these two traditions of healing should cooperate in order to achieve what is called 'holistic' therapy, as both systems are not contradictory, but complementary [51]. Also there is recent evidence of importance of working with traditional healers [52,53] and recommendation that the Mental Health Policies should not be based exclusively on medical (scientific) models, but incorporate larger socio-cultural and religious dimensions [54].

## Conclusion

Improved provision of accessibility to modern primary health care, making psychotropic medications readily available in these set ups, having a good referral system and free provision of allopathic services by the government to the Omani population does not necessarily translate into improved utilization of modern mental health services by adolescents and youth. Males underutilize these services compared to females and need to be aggressively targeted. Cultural issues, traditional belief systems and role of religion are the barriers which need to be integrated for better delivery of mental health services by the government. Decision makers need to bridge the gap between providing exclusive modern allopathic services and the existing non-allopathic approach which is equally utilized and perhaps well acceptable in the Omani society. It is a challenging task, but can help to break the existing barriers in the mental health services. Mental health services for adolescents and youth have direct bearing on their future quality of life, and there can possibly be no good health without good mental health.

## Abbreviations

BMD: Bipolar Mood Disorder; CIDI: World Mental Health-Composite International Diagnostic Interview; DSM IV: Diagnostic and statistical manual of mental disorders, 4^th ^edition; MDD: Major Depressive Disorder; PAPI version: Paper and Pencil Instrument version; WMH-CIDI: World Mental Health - Composite International Diagnostic Interview.

## Competing interests

The authors declare that they have no competing interests.

## Authors' contributions

AAAR conception, design and drafting the manuscript; SHAA revision of manuscript for intellectual content; HAAK, MMM conception, design and acquisition of data; SSJ interpretation of data analysis, drafting the manuscript and revision for intellectual content.
